# Endoscopy-aided retrieval of broken drainage tube after lumbar spine surgery

**DOI:** 10.1186/s12893-022-01655-3

**Published:** 2022-06-02

**Authors:** Shenshen Hao, Shengli Dong, Hongke Li, Shuai Liu, Honglei Chen, Zhifang Zhang

**Affiliations:** Department of Spine, General Hospital of Pingmei Shenma Medical Group, Pingdingshan City, Henan Province China

**Keywords:** Lumbar spine surgery, Broken drainage tube, Removal, Spinal endoscopy

## Abstract

**Background:**

A ruptured drainage tube which remains in the incision is a rare surgical complication. The usual mode of retrieval is to detach the suture and explore the pre-existing incisional wound. However, spinal endoscopy provides an alternative method for successful removal, avoiding the enlargement of the surgical wound.

**Case report:**

A 53-year-old male patient underwent open lumbar spine surgery for lumbar spondylolisthesis between the 5th lumbar and 1st sacral vertebral bodies. Prior to closure, two negative pressure ball drainage tubes were inserted, one of which broke during removal,beneath the fascia. Use of spinal endoscopy enabled the complete removal of the broken drainage tube. Both the original incisional and endoscopic wounds healed well without any sign of infection.

**Conclusions:**

The use of spinal endoscopy to remove the broken drainage tube is an alternative to open the surgical wound and should be took into account.

## Background

Unsuccessful post-operative drainage tube removal is a rare complication. A ruptured drainage tube which remains in the incision is a potential cause of iatrogenic infection [1]. The usual mode of removal involves detachment of the suture and exploration through the incisional wound which is liable to cause secondary injury to the patient and delay incision healing time. Thus, removal is very difficult without making the wound worse.

Spinal endoscopy is a minimally invasive technique widely used in the treatment of lumbar disc herniation. It requires only a small incision to allow visualization of the internal area of interest. Spinal endoscopy may be a suitable approach to facilitating broken drainage tube removal following lumbar spine surgery.

## Case presentation

A ruptured drainage tube was removed from a patient after lumbar spine surgery using spinal endoscopy.

**Fig. 1:**
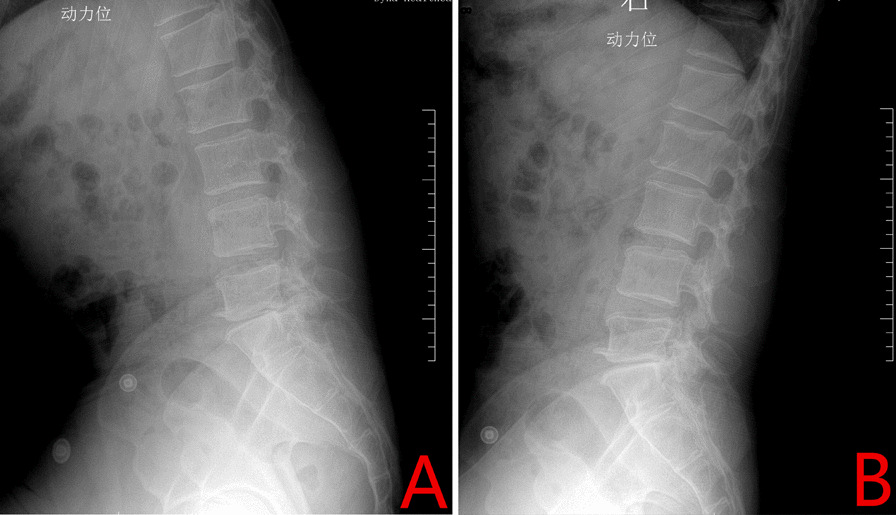
Preoperative radiography. **A** Preoperative flexion X-ray image. **B** Preoperative hyperextension X-ray image. Lumbar spine flexion can be seen to be much reduced prior to the operation with decreased gap between the 5th lumbar and the 1st sacral vertebral bodies

**Fig. 2:**
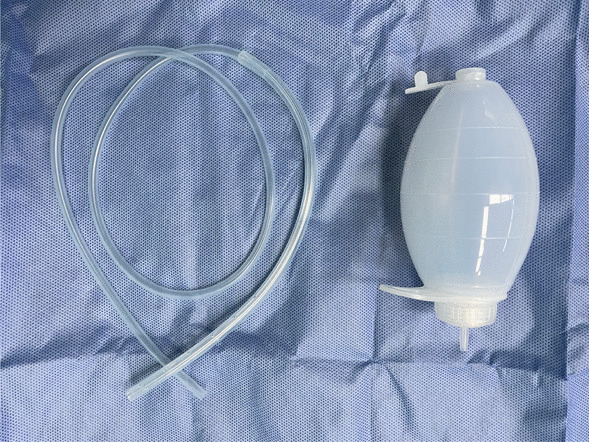
Drainage tube. Silicone drainage tube device used during lumbar surgery

**Fig. 3:**
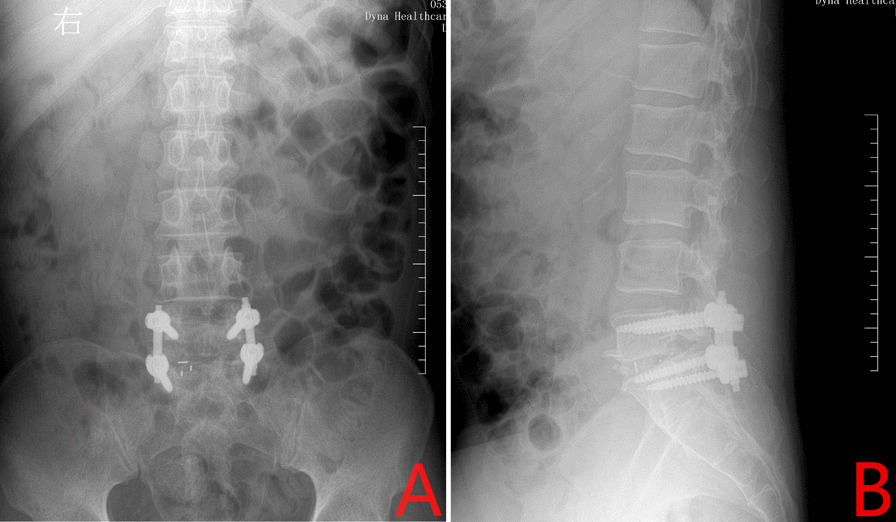
Postoperative radiography. **A** Post-operative anteroposterior X-ray image. **B** Postoperative lateral x-ray image taken on the 3rd postoperative day. The fusion cage and internal fixation device between the 5th lumbar and 1st sacral vertebral bodies can be seen

A 53-year-old male patient underwent open lumbar spine surgery to correct spondylolisthesis between the 5th lumbar and 1st sacral vertebral bodies. The Meyerding classification was degree I (Fig. 1). The operation went smoothly. Prior to closure, two negative pressure ball drainage tubes were inserted with outlets on either side of the incision (Fig. 2) and intradermal suture was used to minimize scar formation. No post-operative infection was present in the incision. Post-operative X-ray reexamination was performed (Fig. 3).

**Fig. 4:**
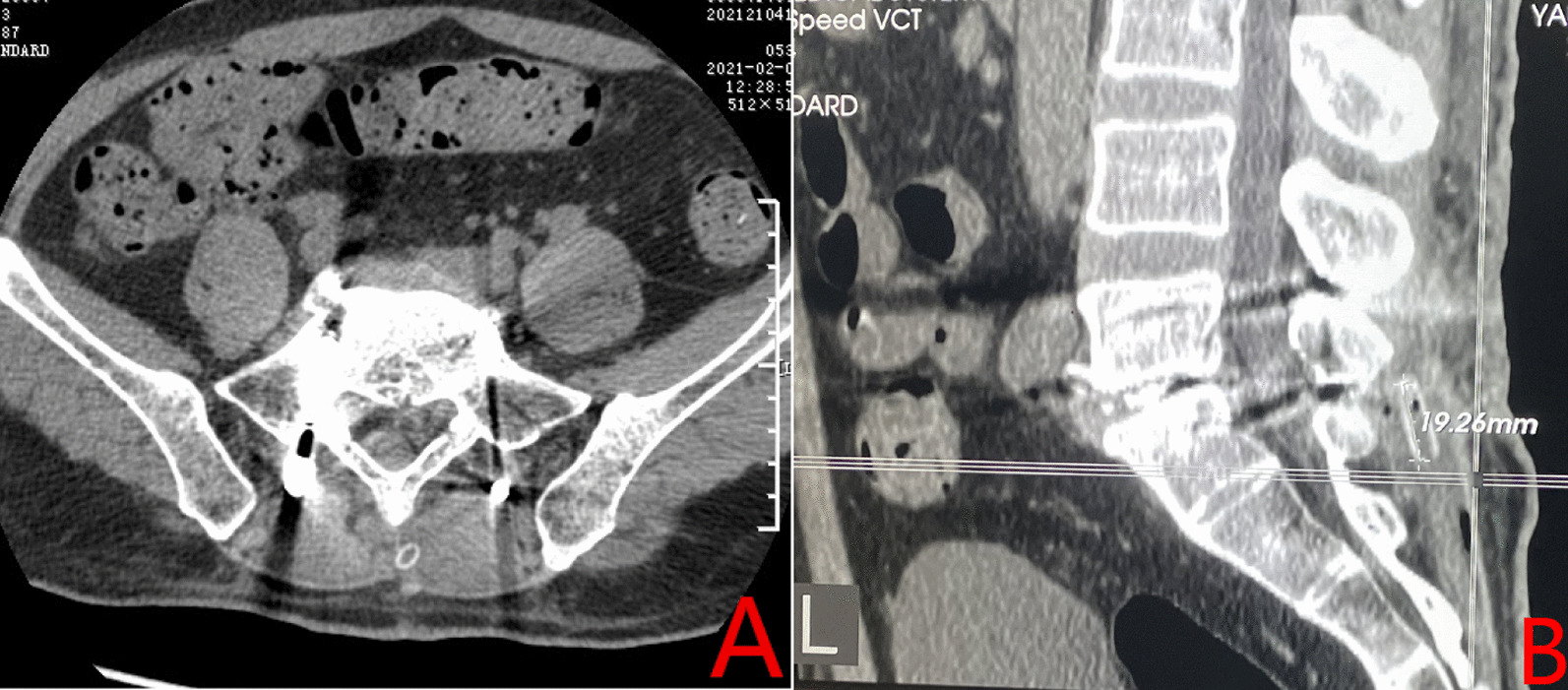
Postoperative computed tomography (CT) scans. **A** Postoperative CT scan. **B** Postoperative CT sagittal scan taken on the 3rd postoperative day. The 2 cm long broken drainage tube can be seen behind the spinous process

The chief surgeon planned to remove the drain without anesthesia but it resisted removal before rupturing. A color Doppler ultrasound examination failed to reveal the broken tube but a CT inspection confirmed its presence in the incision. An approximately 2 cm length was located beneath the fascia near the middle lower incision about 2 cm below the skin’s surface (Fig. 4).

The patient was reluctant to agree to removal of the piece of tube if re-opening of the wound was involved and preferred a minimally invasive approach.

**Fig. 5:**
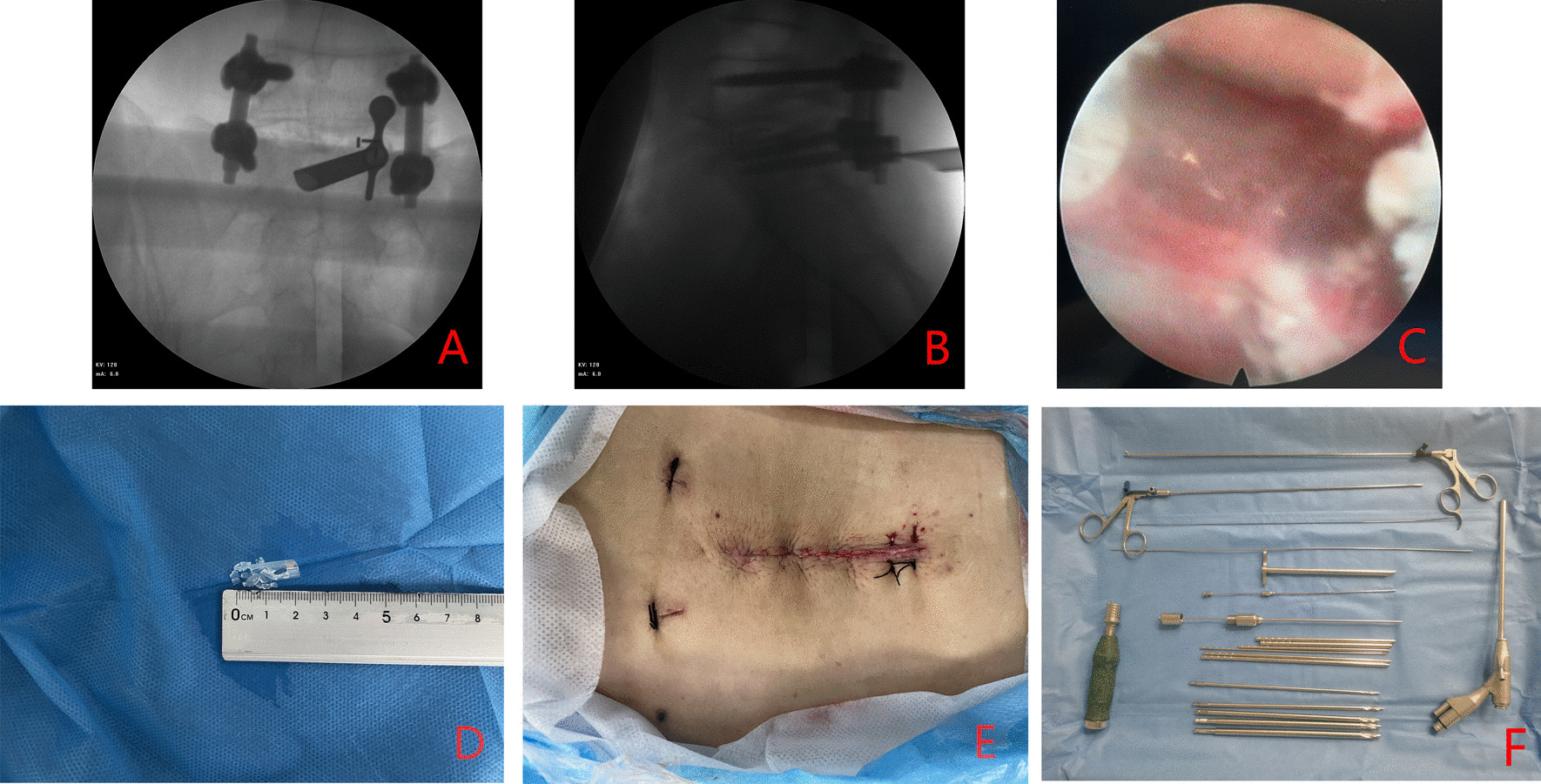
The process of spinal endoscopy for removal of the broken drainage tube. **A** Intraoperative fluoroscopic anteroposterior image during endoscope insertion. **B** Intraoperative fluoroscopic lateral image during endoscope insertion. **C** The broken drainage tube can be visualized via the endoscopic image during the operation. **D** Endoscopy was used to confirm that there was no remnant left and the recovered broken drainage tube was restored to verify its complete removal. **E** The spinal endoscopy incision was sutured with 2 stitches and can be seen at the end of the pre-existing incisional wound. **F** The spinal endoscope used in the operation, the brand is Joimax

Therefore, spinal endoscopy was performed under local anesthesia. The procedure required an incision of less than 2 cm in length, below the original incision and did not necessitate detachment of the fascia layer and the suture. The 2 cm broken drainage tube with sutures running through the double wall could be visualized. It was broken up into small pieces for gradual removal until no residual drainage debris could be visualized (Fig. 5). Two stitches were used to suture the incision.

**Fig. 6:**
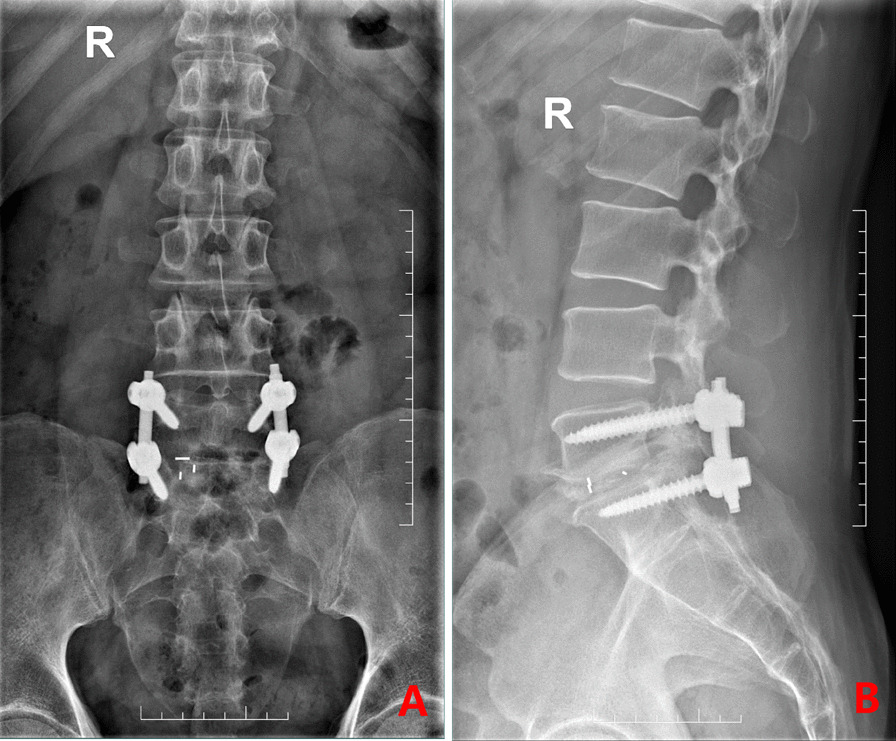
Postoperative follow-up radiography. **A** Postoperative follow-up anteroposterior X-ray image. **B** Postoperative lateral x-ray image taken on follow-up at the 3rd postoperative month. The satisfactory position of the internal fixation device between the 5th lumbar and the 1st sacral vertebral bodies can be seen together with the satisfactory fusion of the two vertebrae

The broken drainage tube was fully removed without increasing the length of the original incision or removing the original sutures. Stitches were removed on 12th day after surgery. No infection or abnormal discomfort developed and the incision healed in two weeks. At 3 months postoperative follow-up, the incision had healed well. X-ray imaging showed a satisfactory location of the internal fixation device and fusion of the 5th lumbar and the 1st sacral vertebral bodies (Fig. 6).

## Discussion and conclusion

**Fig. 7:**
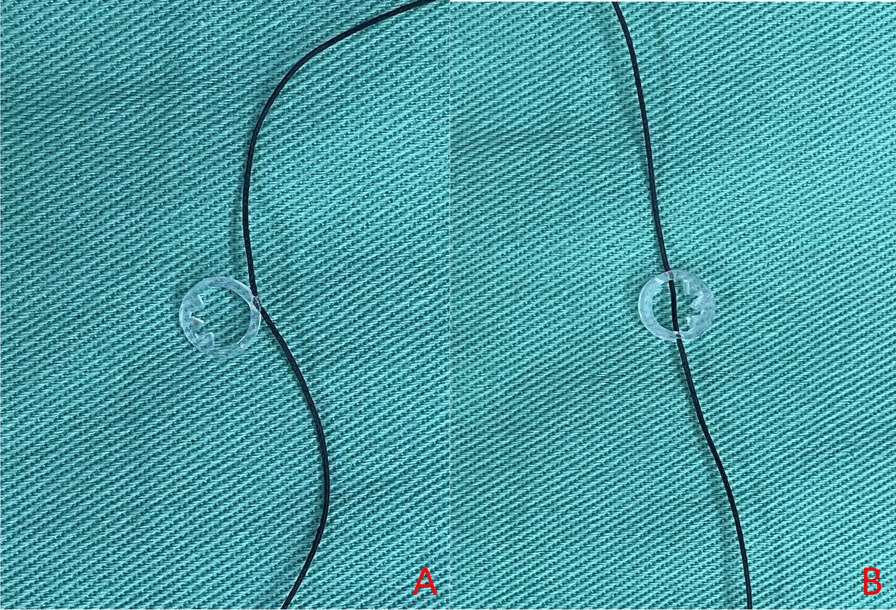
Photos of sutured drainage tubes. **A** Suture through a single layer. **B** Suture through a double layer

Negative pressure ball drainage is often used in spine orthopedics. It is rare for the drainage tube to become entangled or accidentally sutured with tissue during closure. When this does occur, it is likely that the tube will be sutured through a single wall layer, in which case it does not normally break during removal [2], or through double wall layers so breakage is likely took place (Fig. 7). Removal of the broken tube would usually be via detachment of the suture and exploration through the original incision but use of the Kirschner wire tool [2–5] or spinal endoscopy [6] are also possibilities. For tubes left in the patient’s incision, exploration through the surgical incision would be favored but for longer tube pieces located under the fascia layer, the sutures would have to be detached. The current case involved an intradermal suture, re-opening of which would cause secondary injury to the patient and would not be conducive to wound healing.

Spinal endoscopy is a minimally invasive technique used to treat lumbar disc herniation. It allows visualization of the area of interest and requires only a small incision. The suture may remain intact and there is little impact on the healing of the incision [6]. However, the breaking of the tube into small pieces for removal meant that endoscopic visualization was required to ensure that all pieces were removed. The pieces were reassembled and measured after the procedure to ensure that the tube had been completely removed .

Spinal endoscopy technology proved to be a successful approach to removing the broken tube in the current case. However, prevention is the best way to avoid such situations and proper care should be taken over the placement and removal of drainage tubes in the operating theatre. Visualization of the drainage tube should be maintained during stitching of the incision and the length of the tube buried in the incision should be slightly shorter than the length of the incision. These precautions prevent the bending of the drainage tube and reduce the probability of its being accidentally sutured to tissue during wound closure. In addition, any extra side holes should not exceed 1/3 of the tube’s circumference, should not be on the same side nor too close together to avoid weakening the silicone constituent material [7]. During wound closure, the drainage tube may be agitated gently back and forth to ensure that it is not accidentally sutured to tissue. Care should be taken to avoid bending of the tube during this procedure. Furthermore, removal should involve pulling out slowly and gently along the direction of the tube. In case of difficulty, the tube could be rotated clockwise or counterclockwise to facilitate its smooth extraction.

The current case highlights the danger of accidental suturing of a drainage tube. However, the use of spinal endoscopy to remove a broken drainage tube is a viable alternative option to open the original surgical wound. The endoscopic removal in small piece may be dangerous, once a piece of drain can still be there, compared to open removal. This is a potential limitation of the technique. Care should be taken during drainage tube placement and removal to avoid damage and breakage of the tube.

## Data Availability

All data and materials during this study are included in this article.
